# Prevalence of Hepatitis B virus genotypes in HBsAg positive individuals of Afghanistan

**DOI:** 10.1186/1743-422X-8-281

**Published:** 2011-06-07

**Authors:** Sobia Attaullah, Saif ur Rehman, Sanaullah Khan, Ijaz Ali, Sajid Ali, Shahid Niaz Khan

**Affiliations:** 1 Department of Zoology, Islamia College Peshawar (A Public Sector University), University Campus Peshawar 25120, Khyber Pakhtunkhwa, Pakistan; 2 Directorate of Legal Medicine, Kabul, Afghanistan; 3 Molecular Parasitology and Virology Laboratory, Department of Zoology, Kohat University of Science and Technology, Kohat 26000, Khyber Pakhtunkhwa, Pakistan; 4 Institute of Biotechnology and Genetic Engineering, KP University of Agriculture Peshawar, Khyber Pakhtunkhwa, Pakistan; 5 Department of Biotechnology, Kohat University of Science and Technology, Kohat 26000, Khyber Pakhtunkhwa, Pakistan

## Abstract

**Background:**

The structural and functional differences between hepatitis B virus (HBV) genotypes are the mainstay to severity, complications, treatment and possibly vaccination against the virus. This study was conducted to determine the HBV genotypes in HBsAg positive patients of Afghanistan as no such large scale data available previously.

**Methods:**

Two hundred and fourteen HBsAg-positive patients were included in this study. All patients were anti-HCV and anti-HIV negative. All the samples were confirmed for HBV DNA with nested PCR while HBV DNA positive samples were subjected to type specific PCR for HBV genotyping (A-F).

**Results:**

Of the total samples, 168 (78.5%) were males and 46 (21.49%) females, aged ranged between 18 to 71 years. This study demonstrated that genotype D (35.67%) is the predominant genotype circulating in Afghani's population. Genotype C was observed in 32.16% followed by genotype A (19.30%), and genotype B (7.02%) while 6.07% of the individuals were not typed.

**Conclusion:**

This study has shown a heterogeneous distribution of HBV genotypes. Further more, extensive studies are required to investigate genetic and geographical divergence and characteristics of the virus in the country, as no such large sample sized study has been carried out so far in this country.

## Background

Hepatitis B virus (HBV) infects nearly two billion people worldwide [1], cause approximately 600,000 deaths annually due to acute and chronic consequences [2]. HBV is classified into eight genotypes, from A to H, based on an intra-group nucleotide divergence of up to 4.2% of the S-genome sequences or in >8% of the entire genome sequences [1-5], which consists of about 3200 base pairs [5]. These genotypes arise during replication as a result of nucleotide mis-incorporations, in the absence of any proofreading capacity by the viral polymerase [1]. HBV exhibits genetic variability with an estimated rate of 1.4 - 3.2 × 10^-5 ^nucleotide substitution per site per year [6].

A greater demand for genotyping of patient strains of HBV are growing as specific clinical associations with each genotype becomes increasingly apparent [5]. HBV genotypic determination is of particular importance for the study of the detection of the virus's origin, course of evaluating HBV, the severity and activity of liver disease [1,4,5], prognosis and response to antiviral treatment [1,3], patterns of serological reactivity and replication of the virus [1]. Recent studies demonstrated that a particular genotype may affect clinical manifestations during the course of the disease. As patients affected by genotype A have a better prognosis, genotype B enhances the possibility of hepatic malignancy [4], genotype C is associated with faster liver damage than genotype B [2], alanine transaminase level and the index of inflammatory cellular necrosis in genotype C was higher than in genotype B [2], genotype D may develop fulminant hepatitis with high frequency [4,5], may be more associated with liver cirrhosis compared to A [2,5], whereas genotype F was found to be associated with severe infection and young hepatocellular carcinoma development which was found to be more associated with higher mortality rates as compared to other genotypes [2,6].

Afghanistan is landlocked and positioned in the Middle East, a recognized geographical region of southwestern Asia. It's bordered by the countries of China, Pakistan, Iran, Turkmenistan, Uzbekistan and Tajikistan. Over the last three decades of conflict, approximately eight million Afghans have been displaced in neighboring counties. Since 2002, over five million refugees have returned, predominately from Pakistan (3.2 million) and Iran (1.8 million). During conflict and displacement, social networks and services are often disrupted, which may alter sexual behaviors and access to services. In Afghanistan relatively large percentage of young men are in the police force and/or military [7] and are truck drivers, who are frequently away from home for extended periods. Furthermore, refugees may have acquired high-risk behavior, such as injection drug use and unsafe paid sex [8]. The immigration of populations from neighboring countries of high endemic of the infection (particularly Pakistan and Iran) seems to contribute significantly to the rapid modification of epidemiological data of viral hepatitis and may place other communities at risk upon their return [9]. This study was designed to determine and analyze the distribution of HBV genotypes among patients with hepatitis B surface antigen (HBsAg) from Afghanistan. Additional objectives were to determine whether there was an association of HBV genotypes with neighboring countries including Pakistan, Iran and China.

## Materials and methods

### Study Samples

A total of 214 patients with HBsAg positivity were included in this study and written consent was not taken because all the investigations done in this research were routinely for all hepatitis patients, beside no patients identification (name or number) where mentioned in this research. All the blood samples were collected from the Infectious Disease Hospital Kabul, Central Blood Bank Kabul and Central Laboratory Kabul Afghanistan, approved by the ethical committee. Sera were separated and stored at -20°C in Directorate of Legal Medicines Kabul Afghanistan and then transported in cold boxes to the Molecular Parasitology and Virology Laboratory, Department of Zoology, Kohat University of Science and Technology Kohat, Pakistan for further processing. All the samples were analyzed for the detection of HBV DNA and HBV genotyping.

### HBV DNA DETECTION

#### DNA Extraction

DNA was extracted from 100 μl of serum by of GF-1 nucleic acid extraction kit (Vivantas USA) according to the manufacturer procedure with minimal alterations.

#### DNA Amplification

PCR reactions were carried out in a thermal cycler (Nyxtech USA) with 5U*Taq *DNA polymerase (Fermentas USA). The first round of amplification was performed with 5 μl of extracted DNA by using an outer sense primer and an outer antisense primer specific to the surface gene of HBV. Another round of PCR was carried out with inner sense primer and inner antisense primer. Amplified products were subjected to electrophoresis in 2% agarose gel and evaluated under UV transillumination. The 185bp specific amplified HBV DNA product was determined by comparing with the 50-bp DNA ladder (Fermantas USA), used as DNA size marker.

#### HBV Genotyping

HBV genotypes were performed as described by Naito *et al.*, 2001.

## Results

A total of 214 HBs Ag positive individuals with mean age 37.5 ± 11.14 years including 168 (78.5%) men and 46 (21.49%) women age ranged (18 to 71 years) were investigated for HBV DNA presence. Of the total, 138 male and 31 female were confirmed HBV DNA carrier while in 30 male and 15 female HBV DNA was not detected. HBV genotype distribution in 169 HBV DNA positive Afghani patients is shown in Table 1.

**Table 1:** HBV genotype distribution in HBV DNA Positive Afghani Patients (n = 169)

Gender	Genotype				
	
	A	B	C	D	Not typed
Male	30	9	48	45	6
Female	3	3	7	16	2
Total	33(19.53%)	12(7.10%)	55(32.54%)	61(36.09%)	8(4.73%)

**Figure 1:**
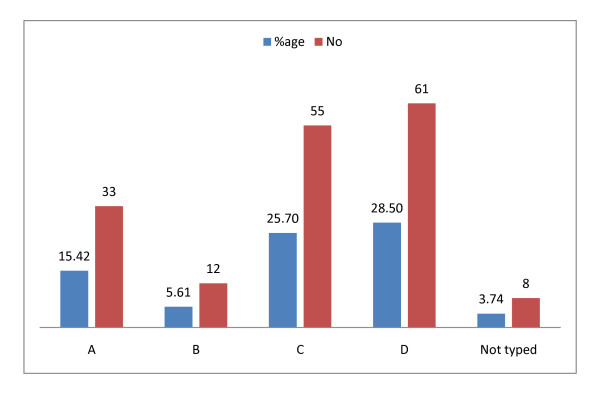
**HBV genotype distribution in HBsAg positive Afghani patients (n = 214)**.

In this study HBV infection in HBs Ag-positive patients (n = 214) were attributed predominantly to viral genotype D constituted 35.67% (n = 61) of the total HBV DNA positive individuals. The prevalence of other HBV genotypes in the study population were like that genotype C was 32.16% (n = 55), genotype B was 7.02% (n = 12), genotype A was 19.30% (n = 33), genotype was not observed in about 3.73% (n = 8) of patients while genotype E, genotype F and mixed genotype was found in none of them Figure 1.

## Discussion

Limited prevalence data for HBV exist for Afghanistan. Overall prevalence of HBV was recorded 6.15% in injecting drug users [10,11], 6.54% in female sex workers [12], 1.53% in obstetric patients and 1.76% in blood donors [9]. HBV genotypes have different biological and epidemiological behavior [5]. Since they influence the activity and outcome of HBV-associated chronic liver disease, as well as the response to antiviral therapies [3-5], their detection and monitoring is more than just academic but also medically significant. Therefore HBV genotyping become a routine exercise in clinical medicine and molecular epidemiology [5]. Since several genotypes HBV are very closely associated with the severity, development of sever liver diseases (cirrhosis and hepatocellular carcinoma) and antiviral therapy. Detection of HBV genotypes is also very important to clarify the pathogenesis, route of infection and virulence of the virus [1].

HBV genotypes and subgenotypes are known to have a distinct pattern of geographic distribution. Mass of evidences showed that genotype A is most common in northwest Europe and the United States, genotypes B and C prevail in Asia, genotype D has a worldwide distribution but predominates in the Mediterranean area, genotype E has been reported from western and South-east Africa, genotype F has been detected in South and Central America [1,3]. To date, the isolation of genotype G has been limited to HBV carriers in France, Germany, United Kingdom, Italy and the United States of America [1] and Genotype H has been described only recently, and its distribution is not understood yet [13].

This study will provide useful information for treatment and management of HBV infection in Afghanistan where there is no such large scale molecular based study of disease has done before. An epidemiologic study with sufficient participants in a wide age range is necessary to address this issue [18]. In this regard the only study published in January 2006 has reported the only genotype among hepatitis B infected patients from Afghanistan was genotype D while other genotypes were not reported [19]. Their study, however, investigated small sample size (n = 12) and therefore, their results cannot be generalized for all Afghanistan population. In our study obtained results for genotype D (35.67%) was similar to other reports from other countries of Middle East area. The most important finding in our results was the nearly equal predominance of the genotype C (32.16%) followed by genotype A that constituted 19.30%. The difference in both studies probably due to the age of the HBV-infected subjects as HBV DNA tends to fluctuate with time [18]. Discrepancy may be correlated to the difference in sensitivity between the two methods used. In other words, sequence analysis provides information only on the majority strain [5]. In this study sensitive technique was used for genotyping, where the distribution of HBV genotype by using PCR technique was also representative for HBV carriers with low viremia and does not have bias even if levels of viremia would be different among genotypes [18].

High HBV prevalence has reported among Afghan refugee populations outside of Afghanistan than that reported among Afghan blood donors [9]. Mass immigration and illegal drug traffic from Pakistan and Iran have all affected epidemiology in Afghanistan [8]. Furthermore, it may be noted that injection drug abuse is common in these countries [10,11] and, thus, could often be the mode of transmission of HBV. IDUs often trade sex for drugs and therefore particularly vulnerable to HBV [8,10]. However, there is a dearth of studies comparing genotypes with these countries. Therefore, present study population provided with a unique opportunity to compare these genotypes.

In Asia, research on genotyping, was initially conducted extensively in Japan and China, therefore, the genotypes of these countries namely B and C were considered as the most prevalent genotypes of Asia. Later on, when research was extended to other countries, predominance of genotype D was found in South Asia and the Middle East including India, Afghanistan and Iran [16]. HBV genotypes show a characteristic geographic distribution with a proposed association with human migration. It is interested to note that Arians firstly colonized to the North of the Caspian Sea, then migrated to Iran, India and Europe. It might be those people who acquired the virus with the genotype D before their migration and then transmitted the virus generation by generation after their migration. That's why the dominant genotype in India, Iran and most part of the Europe is D [17]. History of population migration from the Middle East to Pakistan is also well known, which explains the predominance of genotype D infection in this population [16].

In countries with high levels of immigration, a variety of genotypes are being reported as all of the known genotypes can be found in the Europe and North America [18]. The presence of genotypes A and D also reflected the immigrant origins of the population of Buenos Aires, Argentina which is cosmopolitan city and has received immigration from the Mediterranean area (Afghanistan, Iran, Pakistan, Egypt etc), where genotype D predominates. Thus, we should expect genotype D rather than A to be prevalent. It has been suggested that genotype D may have replaced genotype A in the Mediterranean area [19].

In fact genotyping can help to trace the migration of ancestors as well as the routes of transmission in accidental exposure to HBV [20]. A study of 39 asymptomatic HBV carriers and 103 liver diseases patients from southern China showed circulation of A, B, C, and D genotypes with 78.9% being genotype C [13]. However, in Pakistanis 62% were genotype D, A (14%), C (6%), other genotypes (4%) and recombination (10%). Interestingly, no genotype other than D has been found in Iran. The epidemiological data about HBV genotypes in various Asian countries demonstrated the presence of all seven genotypes, particularly the pre-dominance of genotype D [17]. This close relationship between Afghanistan and neighbouring countries demonstrated a circulation of closely related viruses. Future studies are needed to carry out work on the prevalence of HBV genotypes and sub genotypes across the Afghanistan and compare it to the neighboring countries. At the same time, current crisis in Afghanistan has resulted in an influx of Western military personnel, peacekeepers, humanitarian workers, and journalists [21].

## Conclusions

To be successful, HBV prevention, treatment and vaccination strategies must be based on a sound understanding of the genotypes and epidemiological behavior of the virus, especially in those geographic regions where there no genotype base study has done. It was concluded that HBV genotype demonstrated significant divergence now. In order to shed more light on this subject further investigations are needed to fully unravel the mystery of the prevalence of HBV genotypes all across the Afghanistan to reach a decision concerning their clinical utility.

## Competing interests

The authors declare that they have no competing interests.

## Authors' contributions

SK and SA were involved in the design and literature searching. SR and SA performed lab work. IA and SNK revised critically the manuscript. All the authors read and approved the final manuscript.
